# Lower extremity arterial disease in patients with diabetes: a contemporary narrative review

**DOI:** 10.1186/s12933-018-0781-1

**Published:** 2018-10-23

**Authors:** Mathilde Nativel, Louis Potier, Laure Alexandre, Laurence Baillet-Blanco, Eric Ducasse, Gilberto Velho, Michel Marre, Ronan Roussel, Vincent Rigalleau, Kamel Mohammedi

**Affiliations:** 1 Département d’Endocrinologie, Diabétologie, Nutrition, Hôpital Haut-Lévêque, Avenue de Magellan, 33604 Pessac Cedex, France; 2 Département d’Endocrinologie, Diabétologie, Nutrition, Assistance Publique - Hôpitaux de Paris, Hospital Bichat, DHU FIRE, Paris, France; 3 UFR de Médecine, Université Paris Diderot, Sorbonne Paris Cité, Paris, France; 4 INSERM, UMRS 1138, Centre de Recherche des Cordeliers, Paris, France; 5 Faculté de Médecine, Université de Bordeaux, Bordeaux, France; 6 Département de Chirurgie Vasculaire, CHU de Bordeaux, Bordeaux, France; 7 Fondation Adolphe de Rothschild Hospital, Paris, France

**Keywords:** Ankle–brachial index, Atherosclerosis, Diabetes mellitus, Intermittent claudication, Lower-extremity arterial disease, Peripheral arterial disease, Revascularization

## Abstract

Lower-extremity arterial disease (LEAD) is a major endemic disease with an alarming increased prevalence worldwide. It is a common and severe condition with excess risk of major cardiovascular events and death. It also leads to a high rate of lower-limb adverse events and non-traumatic amputation. The American Diabetes Association recommends a widespread medical history and clinical examination to screen for LEAD. The ankle brachial index (ABI) is the first non-invasive tool recommended to diagnose LEAD although its variable performance in patients with diabetes. The performance of ABI is particularly affected by the presence of peripheral neuropathy, medial arterial calcification, and incompressible arteries. There is no strong evidence today to support an alternative test for LEAD diagnosis in these conditions. The management of LEAD requires a strict control of cardiovascular risk factors including diabetes, hypertension, and dyslipidaemia. The benefit of intensive versus standard glucose control on the risk of LEAD has not been clearly established. Antihypertensive, lipid-lowering, and antiplatelet agents are obviously worthfull to reduce major cardiovascular adverse events, but few randomised controlled trials (RCTs) have evaluated the benefits of these treatments in terms of LEAD and its related adverse events. Smoking cessation, physical activity, supervised walking rehabilitation and healthy diet are also crucial in LEAD management. Several advances have been achieved in endovascular and surgical revascularization procedures, with obvious improvement in LEAD management. The revascularization strategy should take into account several factors including anatomical localizations of lesions, medical history of each patients and operator experience. Further studies, especially RCTs, are needed to evaluate the interest of different therapeutic strategies on the occurrence and progression of LEAD and its related adverse events in patients with diabetes.

## Introduction

Lower extremity arterial disease (LEAD) is a major manifestation of systemic atherosclerosis with severe associated cardiovascular, lower limb and functional complications. It results from a partial or complete obstruction of one or more lower limb arteries. The first known presentation of lower-limb vascular disease was reported in 1831 in a horse that had a lameness thought to be due to femoral artery occlusion of the posterior limb. Similar symptom was described few years later in humans and characterized as an intermittent claudication (IC). Further investigations showed that IC was linked to muscle ischemia, induced by walking, and considered it as a clinical manifestation of LEAD. During the last decades, a large body of data have reported that LEAD was associated with increased risk of non-traumatic lower limb amputation (LLA), cardiovascular disease (CVD), and mortality [1–3]. Nowadays, LEAD has become an emerging public health burden with an endemic progression worldwide resulting from a demographic expansion, population aging and increasing prevalence of tobacco smoking habits, hypertension, dyslipidaemia, and type 2 diabetes [4–6]. Yet, LEAD is particularly frequent in diabetic patients with worse outcomes, especially the risk of LLA, four to five times higher, compared with non-diabetic subjects [1, 2, 7–9]. Despite its severity, LEAD remains less studied than other diabetic vascular complications; and only few randomised controlled trials (RCTs) have dealt with major lower-limb adverse-events as pre-specified endpoints. Therefore, widespread reviews of the literature dedicated to LEAD are scarce, especially in people with diabetes. We present here a comprehensive narrative review of the available literature to describe and synthesize epidemiology, pathophysiology, screening, diagnosis, and therapeutics of LEAD in patients with diabetes.

## Epidemiology and risk factors

### Prevalence and incidence

LEAD affects over 200 millions people worldwide, including 40 millions living in Europe [5]. It is 2–4 times more frequent in people with type 2 diabetes than in the general population [3, 4]. The prevalence of LEAD varies across studies depending to differences in characteristics of the populations including LEAD definition, age, and ethnicity. Usually discovered during the 5th decade of life, the prevalence of LEAD increased exponentially after 65 years of age. In the Action in Diabetes and Vascular Disease: PreterAx and DiamicroN Modified-Release Controlled Evaluation (ADVANCE) trial, the baseline prevalence of LEAD (defined as lower-limb amputation of at least one digit, chronic foot ulceration due to arterial insufficiency, or peripheral revascularization procedure) was estimated at 4.6% [10]. The LEAD prevalence was much higher and may exceed 20% when its definition was based on abnormal ankle–brachial index (ABI) [2, 4, 11]. The prevalence increases also with rising duration of diabetes as shown in the UK Prospective Diabetes Study (UKPDS): 1.2% at diagnosis of diabetes and 12.5% after 18 years of its evolution [12]. In the same manner, different LEAD incidences were reported: 1.2 per 100 patient-years in ADVANCE trial and 3.7 per 100 patient-years in an Australian cohort [2, 13].

### Prognostic and risk factors

LEAD is one of the major causes of diabetic foot. It was present in 49% of patients with diabetic foot in the EURODIALE study, and one-third of participants had both LEAD and infection [14]. Diabetic patients with LEAD, compared with those without LEAD, have also a higher risk of CVD, and cardiovascular and all-cause mortality [2, 10, 11, 15]. The key risk factors are similar to those related to CVD, including age, sex, tobacco smoking, systolic blood pressure, and plasma concentrations of lipids [12, 13, 16]. A recent study has suggested that the leg fat distribution may be used as a potential marker for predicting CVD [17].

Microvascular disease, mainly macroalbuminuria and diabetic retinopathy, have been shown to be independent risk factors for LEAD [13, 16]. Furthermore, a recent large epidemiological study has shown that low glomerular filtration rate and pathological albuminuria were independently associated with excess risk of LEAD [18]. The risk of LEAD may also vary according to differences in region of origin. In ADVANCE study, the incidence of major LEAD was lesser in Asians compared with participants from Eastern Europe or Established Market economies [13]. Despite a higher rate of CVD, people from South Asia, compared with white Europeans, have a lower prevalence of LEAD [19]. The explanation of this paradox has not yet been clearly elucidated, and genetic predisposition to LEAD may be suspected.

## Pathophysiological mechanisms

**Fig. 1:**
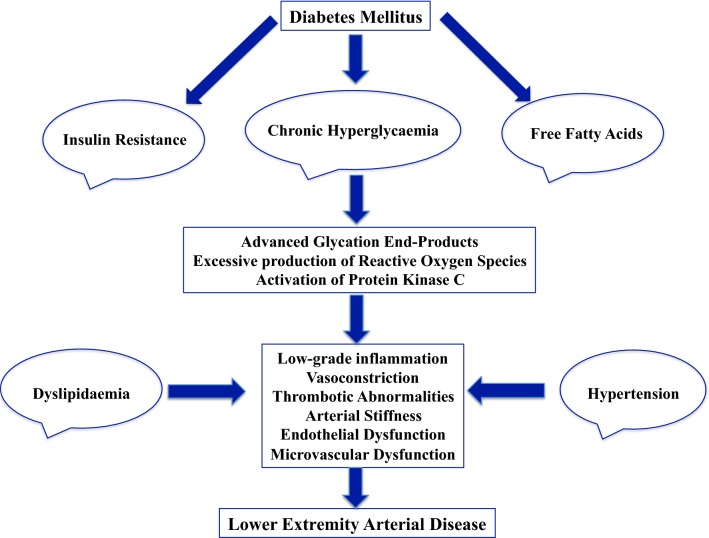
Principal mechanisms involved in the pathogenesis of lower-extremity artery disease in patients with diabetes

Intermittent claudication results from a diminished inflow of oxygen due to a reduced blood flow in the lower limbs during physical activity, which is a consequence of stenosis or obstruction of an artery irrigating the skeletal muscle [20]. Many mechanisms contribute to the development of LEAD, in particular arterial stiffness, thrombotic abnormalities, low-grade inflammation, advanced glycation end-products, and oxidative stress (Fig. 1) [21–23]. Several studies have suggested the development of an acute inflammatory reaction in response to ischemia induced by exercise, with increased release of different biomarkers (thromboxane, interleukin 8, intercellular adhesion molecules, or von Willebrand factor) and vasoconstrictors including endothelin-1 [24]. We have recently reported an independent association between plasma concentrations of tumor necrosis factor-α receptor 1 (TNRF1) and ischemia-modified albumin, inflammatory and redox status biomarkers, and an excess-risk of major LEAD in patients with type 2 diabetes [25]. Interestingly, TNFR1 improves the prediction of LEAD over the traditional risk factors.

Endothelial cells play an important role in vascular biology based on their strategic location between blood and tissues. They secrete many paracrine factors in the vascular wall and its lumen. In pathological setting, endothelial dysfunction induces structural, hemodynamic, and functional vascular abnormalities, altering blood vessels reactivity and relaxation, and generating atherosclerosis [26]. Endothelial dysfunction and increased arterial wall stiffness play an important role in the pathogenesis of LEAD in individuals with diabetes [27, 28].

LEAD has clearly been identified as a common manifestation of atherosclerosis of the large vessels [29]. However, recent studies provided evidence for the implication of microvascular dysfunction in the pathogenesis of macrovascular disease including LEAD [30–32]. An Italian study has shown microvascular histological changes including expansion of the basal membrane and a reduced capillary density in neuro-ischaemic diabetic feet with revascularization requirement [32]. In the ADVANCE study, the baseline history of microvascular disease (defined as the presence of macroalbuminuria (urinary albumin to creatinine ratio > 300 mg/g), requirement of retinal photocoagulation therapy, proliferative retinopathy, macular oedema, or diabetes-related blindness) was independently associated with excess risk of major LEAD during follow-up [13]. Interestingly, microvascular disease was associated with distal LEAD manifestations (lower-limb ulceration or amputation induced by vascular disease) whereas macrovascular disease (defined as the presence of myocardial infarction, stroke, coronary artery bypass graft, percutaneous transluminal coronary angioplasty, or hospital admission for unstable angina or transient ischaemic attack) was linked to proximal presentation (requirement of peripheral revascularization). On the other hand, the baseline history of LEAD was associated with increased risk of advanced diabetic retinopathy, but not with the incidence of end-stage renal disease [10]. Patients with both major LEAD and chronic kidney disease (CKD) may have died before experiencing more advanced kidney endpoints. Of note, a previous study has reported a very high rate (70–80%) of death during 4 years of follow-up in individuals with both LEAD and severe CKD [33].

## Screening and diagnosis

### Clinical examination

The American Diabetes Association (ADA) recommends (Table 1) an initial screening for LEAD based on an exhaustive interview and a clinical examination including a history of decreased walking speed, leg fatigue, claudication, and the palpation of the pedal pulses [34]. Diabetic neuropathy may hide symptoms of LEAD, and should be systematically screened as well. Distal diabetic neuropathy is also involved in medial arterial calcification that leads to incompressible arteries [35, 36]. The clinical presentation of LEAD can be assessed according to Lerich and Fontaine or Rutherford classification [37]. IC and rest pain are the most important signs to be evaluated, though they can be lacking or difficult to attribute exclusively to LEAD. Any deterioration of walking quality or speed must be taken into account as well as fatigue, pain, cramps, discomfort or burns in buttocks, thighs, calfs or feet. Those symptoms are especially suggestive of LEAD when triggered by exercise and quickly relieved with rest. The clinical examination may also contain a careful evaluation of the general aspect of the skin, hairiness, and lower limb temperature. Pulse palpation (distal pedis, posterior tibial, popliteal and femoral arteries), a simple and cheap clinical examination, should be systematically performed in all patients with diabetes [34]. Nonetheless, pulse palpation is not a reliable test; it depends on anatomic variations, physician experience, and examination conditions [38, 39]. Pulse palpation has a weak diagnostic performance [40–42], particularly the dorsal pedis pulse, which can be absent without any vascular abnormalities. However, the absence of both distal pedis and tibial posterior pulses during satisfying exam conditions improve the performance [38, 43].

**Table 1:** Publications of the major international guidelines in screening, diagnosis, and treatment of lower-extremity artery disease

Society	Guidance	Journal	Year	References
American Diabetes Association	Microvascular complications and foot care: standards of medical care in diabetes	*Diabetes Care*	2018	[34]
US Preventive Services Task Force	Screening for peripheral artery disease and cardiovascular disease risk assessment with the ankle–brachial index	JAMA	2018	[46]
American Heart Association & American College of Cardiology	Management of patients with lower extremity peripheral artery disease	Circulation	2017	[84]
European Society of cardiology & European Society for Vascular Surgery	Diagnosis and treatment of peripheral arterial diseases	*Eur Heart J*	2018	[90]

### Ankle–brachial index testing

Ankle–brachial index has emerged as the relatively simple, non-invasive, and inexpensive tool for LEAD diagnosis [44]. The ADA recommends the assessment of ABI as a first line non-invasive test in patients with symptoms or signs of LEAD [34]. It is computed as a ratio of systolic blood pressure at the ankle to the systolic blood pressure in the upper arm. ABI is normal in 1.0–1.4 range, suspicious in 0.9–1 range, and obviously pathologic under 0.9. An ABI over than 1.4 is also considered as abnormal, reflecting calcified and stiffed arteries. ABI was also reported as a marker of CVD and death [45], but the latest US Preventive Services Task Force (USPSTF) recommendation (Table 1) has underlined the lack of evidence supporting the ABI use to screen for LEAD and cardiovascular risk in asymptomatic adult people [46]. The performance of ABI for LEAD screening is particularly inconsistent in people with diabetes [47, 48]. A comprehensive systematic review showed a variable performance: the sensitivity of ABI < 0.9 ranged from 29 to 95% (median at 63%), and its specificity varied between 58 and 97% (median 93%). The addition of ABI > 1.3 did not improve the discrimination. The measurement of ABI is also dependent on operator skills [49]. The performance is particularly affected by the presence of peripheral diabetic neuropathy, medial arterial calcification, and incompressible arteries. In these situations, the toe brachial index may be more effective (pathological if < 0.70). The quality of studies evaluating alternative diagnostic techniques for the screening of LEAD in individuals with diabetes is poor. Otherwise, the toe pressure and the transcutaneous pressure of oxygen (TcPO2) are recommended for the diagnosis of lower limb critical ischemia (see below), and the estimation of the likelihood of wound healing or a requirement of amputation.

### Ultrasound and other imaging methods

The Doppler ultrasound exam is an imaging method with a good LEAD diagnosis performance (sensitivity 93% and specificity 97%) [50]. It is a simple, non-invasive, and an affordable method allowing anatomical and hemodynamic vascular assessments, regardless of medial arterial calcifications, but it remains dependent on the operator experience. The Doppler waveform analysis provides further information; a triphasic waveform reflects a normal hemodynamic state and then the absence of LEAD. The presence of monophasic or biphasic waveforms has a good negative predictive value but her positive predictive value remains less consistent depending on the presence of peripheral neuropathy [51]. Interestingly, a previous study has shown that a semiquantitative score based on the ultrasonographic features of the lower limb arteries may help in the assessment of LEAD across different stages, as well as the evaluation of its associated cardiovascular risk [52]. A recent finding suggested that this score might be better than ABI to screen LEAD [53].

The computed tomography angiography, magnetic resonance angiography and angiography permit a precise topographic diagnosis and are often performed in the pre-operative work-up when large arterial vessels are involved. The topography of LEAD is usually categorized as proximal (from the common iliac to the superficial femoral artery) and distal lesions (from the popliteal to the dorsal pedis artery). The distal localisation has been shown to be more common than the proximal one in patients with diabetes [54].

## Critical limb ischemia

The critical limb ischemia (CLI) is defined as the presence of ischaemic chronic rest pain (> 2 weeks) typically in the forefoot with or without ischaemic lesions or gangrene due to arterial occlusive disease. It is considered as the last stage of LEAD spectrum, with excessively high risk for CVD and death [55]. The CLI is frequent in patients with diabetes, and it may be suspected even in the absence of pain in patients with peripheral diabetic neuropathy. The diagnosis of CLI is confirmed based on one of the following: ABI < 0.4, ankle pressure < 50 mmHg, toe pressure < 30 mmHg or TcPO2 < 30 mmHg. Acute limb ischemia, an emergency condition, needs an urgent diagnosis to evaluate the odds of the limb salvage and to determine the requirement of medical and surgical treatments. The wound ischemia and foot infection (WIFI) classification has been recently recommended by the society of vascular surgery (SVS); it provides a risk stratification based on the severity of the wound, ischemia, and foot infection [56].

## Therapeutic strategies

The management of LEAD in patients with diabetes requires a multidisciplinary team including endocrinologist, vascular surgeon, infectious disease specialist, radiologist, rehabilitation doctor, nurse, and podiatrist. Despite the high macrovascular risk, the based-evidence prevention therapies remain underused in diabetic patients with LEAD compared to their counterparts with coronary or cerebrovascular disease [57, 58]. Therefore, a considerable proportion of patients with diabetes and LEAD remain at increased risk for CVD as well as overall adverse events [2]. A strict control of cardiovascular risk factors is crucial to manage LEAD, and to improve the cardiovascular and the overall prognosis of each patient.

## Anti-diabetic treatment

### Intensive versus standard glucose control

Epidemiological studies and RCTs showed the efficiency of intensive blood glucose control in the reduction of the development and progression of long-term microvascular complications (diabetic nephropathy, retinopathy, and neuropathy) in patients with diabetes [59–61]. However, the benefit of intensive glucose control in the prevention of CVD and death has not been clearly established, and its effect on the risk of LEAD has been rarely addressed in the literature. In the UKPDS trial, each 1% reduction in HbA1c was associated with a 43% decreased risk of major LEAD (amputation or death following a peripheral vascular event) [62]. However, this benefit did not persist during the post-trial observational period of the UKPDS study [63]. In the ADVANCE trial, the incidence of major LEAD (lower-limb ulceration, amputation, revascularization requirement, or death induced by peripheral arterial disease) was comparable among randomized study arms (intensive versus standard glucose control) [13, 61]. A recent systematic review and meta-analyses (with a low level of evidence) displayed 35% reduction of LLA risk in patients with type 2 diabetes assigned to intensive glycaemic control compared with those assigned to less intensive strategy, but no effect was observed on ischemic disease [64].

### Insulin-sensitizing versus insulin-providing therapy

The PROspective pioglitAzone Clinical Trial In macroVascular Events (PROactive) trial showed a non-significant association between use of pioglitazone, an agonist of peroxisome proliferator-activated receptor γ (PPAR γ), and a higher risk of leg revascularization, compared with placebo [65]. However, the post hoc analyses of the Bypass Angioplasty Revascularization Investigation in Type 2 Diabetes (BARI-2D) trial displayed lower incidence of LEAD (new low ABI ≤ 0.9, lower-extremity revascularization or LLA) among patients assigned to insulin-sensitizing therapy (metformin or thiazolidinedione) compared with those assigned to insulin-providing therapy (sulfonylureas, repaglinide, nateglinide or insulin) [66]. Furthermore, another observational study has shown that the use of metformin was associated with a lower prevalence of below-the-knee arterial calcification [67].

### New anti-diabetic agents

After concerns about the cardiovascular safety of some anti-diabetic drugs, the US Food and Drug Administration (FDA) implemented a guidance statement in 2008 recommending cardiovascular safety trial of each new anti-diabetic agent. Thus, several RCTs were conducted worldwide and demonstrated the non-inferiority of some new inhibitors of dipeptidyl peptidase 4 (DPP-4) or glucagon-like peptide-1 (GLP-1) receptor agonists, compared with placebo in patients with type 2 diabetes [68–71]. Interestingly, other trials have shown cardiovascular benefit of some GLP-1 receptor agonists (liraglutide, semaglutide, and albiglutide) or sodium glucose co-transporter 2 (SGLT2) inhibitors (empagliflozin and canagliflozin) [72–76]. In contrast to cardiovascular and cerebrovascular endpoints, LEAD was not fully investigated in these studies. Although, Marso et al. reported in the SUSTAIN-6 trial, that participants treated by semaglutide, a prolonged action GLP-1 receptor agonist, had a significant 35% lower risk of coronary and peripheral revascularization, but with no specific data dedicated to lower-limb procedures [73]. A recent post hoc analysis of the liraglutide effect and action in diabetes: evaluation of cardiovascular outcome results (LEADER) trial displayed a fewer LLA rate among patients with diabetic foot assigned to liraglutide, compared with those assigned to placebo [77]. This difference seemed to be driven mainly by major amputation rather than minor amputation, but there was no difference between study arms in diabetic foot requiring peripheral revascularization. The incidence of LEAD was similar among study arms (exenatide versus placebo) in the Exenatide Study of Cardiovascular Event Lowering (EXCEL) trial [78]. Notably, the Canagliflozin Cardiovascular Assessment Study (CANVAS) trial has shown a twofold higher risk of LLA in participants assigned to canagliflozin, compared with those assigned to placebo [75]. This increased risk of amputation has been mainly driven by vascular disease, while the rate of diabetic neuropathy seemed to be lower in the canagliflozin group than in the placebo arm (CANVAS Program Collaborative Group, the 53rd Annual Meeting of the European Association for the Study of Diabetes, Lisbon, 15 September 2017; https://www.easd.org/programme-glance.html). The pathophysiological mechanisms likely to explain the high risk of LLA associated with canagliflozin in the CANVAS trial have not yet been established. It remains unclear if the risk of amputation is a class effect for all SGLT-2 inhibitors. While some studies suggested association with different SGLT-2 inhibitors and increased risk of LLA [79–81], the secondary analyses of the EMPA-REG OUTCOME trial have shown a similar incidence of LLA in empagliflozin versus placebo group [82]. Furthermore, a recent large real-life study conducted in the USA did not show association between SGLT2 inhibitors and the risk of LLA [83].

## Antihypertensive drugs

The American Heart Association (AHA) and the American College of Cardiology (ACC) recommend antihypertensive treatment in patients with LEAD to decrease cardiovascular events and stroke (Table 1) [84], but the benefit-risk of each antihypertensive class in term of LEAD-related events has not yet been fully investigated even in the general population [85]. The relationship between blood pressure and LEAD is not simple, and may be U-shaped in the general population [86]. In the type 2 diabetes setting, the risk of LEAD increased with rising systolic blood pressure and decreasing diastolic blood pressure, and is particularly associated with growing pulse pressure [13], which is known as a surrogate of arterial stiffness [87]. Interestingly, each 10 mmHg decrease of systolic blood pressure was associated with 16% reduction of LEAD risk in the observational period of the UKPDS study [88]. The post hoc analyses of the Veterans Affairs Diabetes (VADT) trial have also shown a reduction of the ischemic LLA rate in participants with systolic blood pressure < 140 versus ≥ 140 mmHg [89]. However, the ADVANCE trial has not reported any LEAD benefit related to perindopril/indapamide treatment, compared with placebo [13].

## Lipid lowering drugs

### Statin therapy

The European Society of Cardiology (ESC) and the European Society for Vascular Surgery (ESVS) recommended targeting serum low-density lipoprotein cholesterol (LDL-C) less than 1.8 mmol/L (< 70 mg/dL) or decreased by ≥ 50% if the initial value is between 1.8 and 3.5 mmol/L (70 and 135 mg/dL) for all patients with LEAD (Table 1) [90]. Although the lack of specific evaluations of the effects of lipid-lowering drugs on the occurrence of LEAD-related endpoints, observational studies and few RCTs provide evidence for reductions of cardiovascular events and all-cause mortality in patients using statins [91–93]. In the Reduction of Atherothrombosis for Continued Health (REACH) registry, statin use was associated with a 17% decrease in adverse cardiovascular events rates among individuals with LEAD, without heterogeneity regarding diabetes status [94]. Other studies have also suggested that statin may reduce the LLA incidence and improve walking distance in patients suffering for IC [95–98].

### Fibrate therapy

The use of Fenofibrate failed to reduce macrovascular events in participants with type 2 diabetes in the Action to Control Cardiovascular Risk in Diabetes (ACCORD) and the Fenofibrate Intervention and Event Lowering in Diabetes (FIELD) trials [99, 100]. However, secondary analyses of the FIELD trial displayed a 36% reduction in the risk of LLA (a pre-specified tertiary endpoint) in participants assigned to fenofibrate, compared with those assigned to placebo [101]. This protection has been especially driven by decreased risk of minor amputation without known large-vessel disease rather than amputation with large-vessel lesions.

### PCSK9 inhibitors

Despite the availability of effective drug therapies that reduce LDL-cholesterol, CVD remains an important cause of mortality and morbidity. Therefore, additional LDL-cholesterol reduction may be warranted, especially for patients who are unresponsive to, or unable to take, existing LDL-cholesterol reducing therapies. Proprotein convertase subtilisin/kexin type 9 (PCSK9) is a serin protease with effect on the LDL receptor cycle leading to its degradation and therefore inhibition of continuing LDL-cholesterol clearance from the blood. This path is the target of newly developed lipid-lowering drugs, PCSK9 inhibitors, monoclonal antibodies leading to further LDL-cholesterol decrease, with reducing CVD risk, but not cardiovascular or all-cause mortality [102]. The Further Cardiovascular Outcomes Research with PCSK9 Inhibition in Subjects with Elevated Risk (FOURIER) trial has shown that evolocumab, versus placebo, reduced LDL-cholesterol and adverse cardiovascular events among patients with elevated cardiovascular risk on statin therapy [103]. Evolocumab decreased cardiovascular endpoints reliably in participants with and without diabetes at baseline [104]. The use of Evolocumab was also associated with 42% reduction of LEAD-related events (acute limb ischemia, major amputation, or urgent peripheral revascularization for ischemia) with consistent effects in those with or without known LEAD at baseline [105]. There was a consistent relationship between lower achieved LDL-cholesterol and reduced risk of LEAD-related events.

## Antiplatelet and anticoagulation therapies

### Antiplatelet therapy in symptomatic or asymptomatic LEAD

Antiplatelet drugs are advised in all patients with symptomatic LEAD or having undergone previous vascular revascularization, to reduce both CVD and peripheral vascular event. The ESC and the ESVS guidelines did not recommend antiplatelet therapy in subjects with asymptomatic LEAD (Table 1) [90]. In the general population, aspirin, compared with placebo, did not reduce vascular events among participants with asymptomatic LEAD [106]. Furthermore, the prevention of progression of arterial disease and diabetes (POPADAD) trial did not provide evidence to support the use of aspirin or antioxidant agents in the primary prevention of macrovascular events (including amputation above the ankle for critical limb ischaemia) in 1276 diabetic patients with asymptomatic LEAD [107].

### Single antiplatelet therapy

The meta-analyses from the Antithrombotic Trialists Collaboration group showed that aspirin (or another oral antiplatelet drug) was protective in different high vascular risk populations, including those with LEAD [108]. However, some data might encourage the use of clopidogrel rather than aspirin in LEAD condition, especially in people with diabetes. A meta-analysis of 18 RCTs comparing aspirin to placebo in 5269 patients with symptomatic or asymptomatic LEAD did not show a significant reduction in cardiovascular adverse events, except for non-fatal stroke considered individually as a secondary endpoint [109]. No significant association was observed between aspirin treatment and the other secondary outcomes including all-cause or cardiovascular mortality, myocardial infarction, or major bleeding. The aspirin in patients at risk of ischaemic events (CAPRIE) trial, involving 20% of participants with diabetes, has displayed reduction of the risk of LEAD-related events in participants assigned to clopidogrel 75 mg compared with those assigned to aspirin 325 mg [110]. Finally, treatment by Ticagrelor (90 mg twice daily) has not been shown to be superior to clopidogrel (75 mg once daily) for the reduction of cardiovascular or limb events in 13,885 participants (38% of whom had diabetes) with symptomatic LEAD [111].

### Dual antiplatelet therapy

No evidence exists for any benefit related to a dual antiplatelet therapy in patients with LEAD. In the post hoc analyses of the Clopidogrel for High Atherothrombotic Risk and Ischemic Stabilization, Management, and Avoidance (CHARISMA) trial, dual therapy (clopidogrel and aspirin) did not provide further vascular protection over aspirin alone in LEAD patients (36% with diabetes), except for the risk of myocardial infarction and hospitalization for ischaemic events [112]. This modest beneficial effect of dual therapy was counterbalanced by an increased risk of bleeding. Some groups suggest the use of dual antiplatelet therapy for at least 1 month after endovascular therapy for LEAD with a stent implantation irrespective of its type [90].

### Anticoagulant therapy

Anticoagulation strategy is currently advisable in the presence of its traditional indication (e.g. atrial fibrillation), although new drugs have provided encouraging findings for LEAD-related events. The cardiovascular outcomes for people using anticoagulation strategies (COMPASS) trial showed that rivaroxaban, an oral factor Xa inhibitor, plus aspirin was associated with fewer adverse cardiovascular events, but more major bleeding events versus aspirin alone [113]. The rivaroxaban treatment was also associated with reduced major limb events in patients with carotid or lower-limb peripheral artery disease. This benefit was reliable in participants with or without diabetes at baseline [114]. The Trial to Assess the Effects of SCH 530348 in Preventing Heart Attack and Stroke in Patients With Atherosclerosis (TRA2°P-TIMI 50) revealed that Vorapaxar, a novel antagonist of protease-activated receptor-1, reduced the rates of hospitalization for acute limb ischemia and peripheral artery revascularization, but did not reduce the risk of cardiovascular death, myocardial infarction, or stroke in patients with stable atherosclerotic vascular disease and LEAD [115]. The Vorapaxar use was also associated with an increased risk of bleeding. These new therapies may improve the management of patients with LEAD, but the excess risk of bleeding should be seriously considered.

## Multifactorial intervention therapy

The Steno2 trial has compared a multifactorial intervention versus conventional treatment in patients with type 2 diabetes and microalbuminuria [116]. During the 7.8-year in-trial period, Gæde and co-workers observed a fewer number of LLA (7 versus 14) and surgical procedures for peripheral atherosclerotic artery disease (6 versus 12) in participants assigned to multifactorial versus conventional therapy. These benefits persisted during the 5.5-year observational post-trial period [117]. On the other hand, no difference was observed in term of lower limb vascular events (amputation or revascularisation) in the Japan Diabetes Optimal Treatment study for 3 major risk factors of cardiovascular diseases (J-DOIT3) that compared the effectiveness and safety of a multifactorial intervention for control of glucose, blood pressure, and LDL cholesterol, versus strategy based on the ongoing Japanese guidelines in patients with type 2 diabetes [118].

## Other vasodilator therapies

Some vasodilator agents may be used to relieve intermittent claudication and increase walking distance in patients with LEAD. The cilostazol, a selective inhibitor of the phosphodiesterase III, is the most studied drug; its benefit is modest with no evidence for vascular protection. In a comprehensive meta-analyses, cilostazol improved walking distance with no relevant cardiovascular effect or improvement in quality of life [119]. This vasodilator is responsible for some adverse effects including headaches, vertigo, palpitations, and diarrhea. The cilostazol also acts as an antiplatelet drug and therefore must be associated cautiously with other antiplatelet drugs or anticoagulant agents [120]. Naftidrofuryl, a peripheral vasodilator, also improved significantly the walking distance [121]. Finally, Buflomedil, a vasoactive agent, has only a small benefit in term of IC, and has been linked to some safety concerns including lethal and non-lethal neurologic and cardiovascular events in cases of accidental and voluntary overdoses [122].

## Innovating treatment

### Growth factor therapy

Some data suggested a relationship between circulating levels of growth factors and the development of LEAD [123]. The therapies using growth factors, delivered directly (as recombinant proteins), or indirectly (e.g. by viral vectors or DNA plasmids encoding these factors), have been tested in LEAD with contrasting findings. The Efficacy and Safety of XRP0038/NV1FGF in Critical Limb Ischemia Patients With Skin Lesions (TA-MARIS) trial did not show relevance of non-viral 1 fibroblast growth factor in the reduction of death or LLA in 520 participants (53% with diabetes) with critical limb ischemia unable for revascularisation [124]. A recent meta-analysis of 14 RCTs investigating fibroblast, hepatocyte, and vascular endothelial growth factors did not support their use in patients with LEAD in term of death, major amputation, or IC. However, these factors may improve haemodynamic measurements and decrease the risk of minor amputation [125].

### Stem cell therapy

Some studies have tested stem cell therapy in patients with LEAD with encouraging results, although the lack of definitive evidences. A recent systematic review of the literature and a meta-analysis have shown that autologous cell therapy reduced the risk of LLA and rest pain, improved wound healing, and increased ABI and TcPO2 in patients with LEAD who were ineligible for surgical or percutaneous revascularization [126]. Interestingly, the benefit of cell therapy on LLA rate was higher in trials with a high prevalence of diabetes at baseline. Cell therapy was not associated with severe adverse events. All benefits were especially observed in non-randomized studies and cell therapy versus standard care RCTs. However, these associated benefits were not significant in placebo-controlled randomized trials, and disappeared in RCTs with a low risk of bias. Further high-quality placebo-controlled randomized trials are needed to confirm the safety and the efficiency of autologous cell therapy in patients with LEAD. Of note, a recent placebo-compared RCT (Patients With Intermittent Claudication Injected With ALDH Bright Cells (PACE)) did not support the use of cell therapy in patients with LEAD [127]. PACE trial has not shown improvement in peak walking time, collateral count, peak hyperaemic popliteal flow, and capillary perfusion in patients with LEAD treated by autologous bone marrow-derived aldehyde dehydrogenase bright cells.

### Other innovating procedure

Remote ischaemic conditioning (RIC), involving repeated applications of short periods of limb ischemia over days or weeks, may improve endothelial function, skin microcirculation, and regulates the inflammatory response. Some data suggested that repeat RIC may boost healing of ischaemic diabetic foot [128].

## Lifestyle management

### Smoking cessation

Tobacco smoking including second-hand smoke has been highlighted as one of the three leading risk factors for global disease burden worldwide [129]. It is an independent risk factor for LEAD [12, 13, 16], and has been shown as a significant predictor for worse outcomes including peripheral events and death in patients with LEAD undergoing infra-inguinal bypass [130]. Tobacco smoking may induce prothrombotic and atherogenic abnormalities, and increase the risk of acute myocardial infarction, sudden cardiac death, stroke, aortic aneurysm, and LEAD [131, 132]. Both passive and active smoking cessation is required in all patients with peripheral arterial disease including LEAD. Health authorities should adopt effective public health policies limiting tobacco use, especially in low- and middle-income countries.

### Exercise training

The exercise training improved walking ability, and distances, as well as physical function, vitality and general health [133]. However, the exercise training did not increase ABI, or reduce the risk of LLA, cardiovascular events, or mortality. Some data showed significant improvement in maximal and pain-free walking distance in participants assigned to supervised exercise therapy, compared with non-supervised exercise therapy regimens [134]. No significant difference was observed in term of quality of life parameters between the two exercise programs.

### Nutrition therapy

Healthy diet might help to achieve and maintain body weight goals, reach individualized glycaemic, blood pressure, and lipid targets, and delay or prevent diabetic complications, especially microvascular disease [135]. Previous studies suggested the influence of nutrient quality on the prevalence of LEAD [136, 137]. A systematic review suggested that Omega‐3 fatty acids might have modest haematological benefit in people with IC, but no improvement in walking distance, ABI, angiographic measurements, or quality of life [138]. Interestingly, a Spanish trial has suggested that the Mediterranean diet supplemented with extra-virgin olive oil or nuts, compared with a low-fat diet, was associated with a lower risk of LEAD [139].

## Surgical or endovascular revascularization

Contrasting observations were reported in terms of perioperative outcomes in patients with diabetes undergoing revascularization [140–144]. Recent studies have shown similar peri- and post-operative mortality in patients with diabetes, compared with those without diabetes, but diabetic patients had a higher risk of incomplete wound healing and major amputation, a prolonged length of hospital stay and more frequent readmission [143, 144]. Surgical revascularization provides good long-term patency, although with a longer hospital stay and increased risk for perioperative complications and mortality, when compared with endovascular procedure [145]. The development of new techniques last decades has encouraged the implementation of endovascular therapy in patients with LEAD. The different options of revascularization depend on several factors including anatomical location, extension, and length of arterial lesions; general health condition of each patient and comorbidities; as well as centre and surgeon experience. The endovascular revascularization may be a good strategy for short (< 5 cm) stenosis or occlusion of iliac arteries, providing a good long-term patency [146]. Whereas, a hybrid procedure (endarterectomy or bypass at the femoral level combined with endovascular therapy) may be indicated for ilio-femoral lesions [147]. Aorto-femoral bypass is the first line strategy in aorto-iliac occlusions in patients who fit for surgery [148], while an endovascular procedure should be considered in long or bilateral lesions in patients with severe comorbidities [145, 149]. An endovascular revascularization may be also considered as a first strategy for aorto-iliac occlusive lesions if done by an experienced team without compromising subsequent surgical options [150]. In femoro-popliteal stenosis/occlusions < 25 cm, an endovascular revascularization may be considered as the first-line therapy, and a primary stent implantation has been associated with further morphological benefits [151, 152]. If the occlusion/stenosis is more than 25 cm, surgical bypass may be an appropriate option with a better long-term patency, especially when using the great saphenous vein. The infra-popliteal artery disease is a common LEAD presentation in patients with diabetes. The ESC and ESVS recommends endovascular therapy as first choice in infra-popliteal artery disease with stenotic lesions and short occlusions, while bypass with an autologous vein gives may be discussed for long occlusions (Table 1) [90]. However, endovascular therapy can be tried in patients with long occlusions if the surgical risk is judged as high, or in the absence of autologous vein. The angiosome concept, targeting the ischaemic tissues, may also be considered.

## Conclusion

LEAD is one of the most severe conditions seen in patients with diabetes. It leads to excess risk of death, CVD, and limb loss, and is responsible for disabilities and an important socio-economical burden. The diagnosis strategy has been better codified now focusing in patients with evocative symptoms of LEAD, far from the worthless and expensive universal screening in asymptomatic patients. Anti-diabetic, anti-hypertensive, lipid-lowering and antiplatelet medications may improve the cardiovascular prognosis of patients with LEAD, but few has been done to test their benefits to reduce the occurrence and the progression of LEAD as well as lower-extremities adverse events. High quality studies are required to advance the knowledge in pathophysiology and natural history of LEAD, and to evaluate different aspects of its management.

## Abbreviations

ABI: Ankle–Brachial Index
ACC: American College of Cardiology
ACCORD: Action to Control Cardiovascular Risk in Diabetes
ADA: American Diabetes Association
ADVANCE: Action in Diabetes and Vascular Disease: PreterAx and DiamicroN Modified-Release Controlled Evaluation
AHA: American Heart Association
BARI-2D: Bypass Angioplasty Revascularization Investigation 2 Diabetes
CANVAS: Canagliflozine Cardiovascular Assessment Study
CAPRIE: Clopidogrel versus Aspirin in Patients at Risk of Ischaemic Events
CHARISMA: Clopidogrel for High Atherothrombotic Risk and Ischemic Stabilization Management, and Avoidance
CKD: Chronic Kidney Disease
CLI: Critical Limb Ischemia
COMPASS: Cardiovascular outcomes for people using anticoagulation strategies
CVD: CardioVascular Disease
DPP-4: DiPeptidyl Peptidase 4
EMPA-REG OUTCOME: Empagliflozin cardiovascular outcome event trial in type 2 diabetes mellitus patients—removing excess glucose
ESC: European Society of Cardiology
ESVS: European Society of Vascular Surgery
EXCEL: Exenatide Study of Cardiovascular Event Lowering
FDA: Food and Drug Administration
FIELD: Fenofibrate Intervention and Event Lowering in Diabetes
FOURIER: Further cardiovascular outcomes research with PCSK9 Inhibition in subjects with elevated risk
GLP-1: Glucagon-Like Peptide-1
IC: Intermittent Claudication
J-DOIT3: Japan Diabetes Optimal Treatment study for 3 major risk factors of cardiovascular diseases
LEAD: Lower Extremity Arterial Disease
LEADER: Liraglutide effect and action in diabetes: evaluation of cardiovascular outcome results
LLA: Lower Limb Amputation
PACE: Patients with intermittent claudication injected with ALDH bright cells
PCSK9: Proprotein Convertase Subtilisin/Kexin type 9
POPADAD: Progression of arterial disease and diabetes trial
PPAR: Peroxisome proliferator-activated receptor
PROactive: pROspective pioglitAzone Clinical Trial In macroVascular Events
RCT: Randomized Clinical Trial
REACH: Reduction of atherothrombosis for continued health registry
RIC: Remote Ischaemic Conditioning
SGLT2: Sodium GLucose co-Transporter 2
SVS: Society of Vascular Surgery
TA-MARIS: Efficacy and Safety of XRP0038/NV1FGF in Critical Limb Ischemia Patients with Skin Lesions
TNRF1: Tumor Necrosis Factor-α Receptor 1
TRA2°P-TIMI 50: Trial to Assess the Effects of SCH 530348 in Preventing Heart Attack and Stroke in Patients With Atherosclerosis
UKPDS: UK Prospective Diabetes Study
USPSTF: US Preventive Services Task Force
VADT: Veterans Affairs DiabeTes
WIFI: Wound Ischemia and Foot Infection
